# 

**Published:** 1904-10-15

**Authors:** 


### NOTICE TO CORRESPONDENTS.

All MSS., Letters, Books for Review, and other matters intended for the Editor should be addressed The Editor, "The Hospital," The Hospital Buildings, 28 & 29 Southampton Street, Strand, W.C.

The Editor cannot undertake to return rejected MS., even when accompanied by stamped directed envelope.

**Notice to Advertisers and Subscribers.**

All Advertisements, Orders for Copies of The Hospital, and Business Communications should be addressed to **THE MANAGER (Not to the Editor),** The Hospital, 28 & 29 Southampton Street, Strand, London, W.C.

The Cost of Subscription, post free, is as follows:—Per week, 3½d.; per quarter, 3s. 3d.; per half-year, 6s. 6d.; per annum, 13s. Foreign and Colonial:—Per annum, 19s. 6d., payable, in all cases, in advance.

**NOTICE.—Vol. XXXV. of "The Hospital" (October 1903 to March 1904), in handsome cloth binding, is now on sale at the office of this Journal, price 10s. 6d. Cases for binding the Numbers contained in this Volume 2s. Index to Vol. XXXV., 3½d., post free.**

**The Monthly part for October is now ready. Price 1s., by post, 1s. 3d.**

### Scraps and Gleanings.

Mr. William Blake has resigned the office of Secretary to the Gorleston Cottage Hospital, after 17 years' zealous service. Until 1902 he was chairman and secretary. The Hospital Board much regret that, as he is about to live in North, Wales they must lose his services.

The Sherwood Forest Sanatorium for Consumption, which is in connection with the Nottingham and Notts Association for the Prevention of Consumption, is to be extended. A new ward, built to memorialise the late Lord Edward Manners, is in course of construction, but in order to utilise this an extension of the administrative buildings is necessary.

### Medical Appointments.

W. R. Alway, M.D., Toronto, Clinical Assistant, Chelsea Hospital for Women. A. W. Anderson, M.B., Ch.B.St.And., Junior House Surgeon, Infirmary for Children, Liverpool. R. D. Attwood, M.R.C.S., L.R.C.P., Resident Assistant Medical Officer of the Southwark Union Infirmary. H. W. Bowen, F.R.C.S., L.R.C.P., Assistant Surgeon, Royal Ear Hospital, Soho. R. Sturgeon Cocke, M.R.C.S., L.R.C.P., Assistant Surgeon, Royal Ear Hospital, Soho. T. Coogan, M.B., Ch.B.Vict., Junior Resident Medical Officer, Withington Workhouse Hospital. A. H. Judson, M.D.Toronto, Clinical Assistant, Chelsea Hospital for Women. Miss Ethilde B. Meakin, M.B., B.S.Lond., Physican-in-charge, Victoria Hospital for Women, Calcutta. A. Logan Murison, M.R.C.S., L.R.C.P., Assistant Surgeon, Royal Ear Hospital, Soho. H. Reginald Pring, M.R.C.S., L.R.C.P., L.D.S.Eng., Assistant Dental Surgeon to the National Dental Hospital. James E. H. Sawyer, M.A., M.D.Oxon., M.R.C.P., Pathologist, General Hospital, Birmingham. J. Hay Shepherd, M.B., Ch.B., M.A.Aber., Senior House-Surgeon, Infirmary for Children, Liverpool. J. H. L. Tylecote, M.B.Vict., Medical Officer and Public Vaccinator for Cullingworth District of the Keighley Union.

Edinburgh Royal Infirmary.—The following appointments were confirmed on September 26th:—Clinical Tutors for the ensuing winter and summer sessions—James Burnet, M.A., M.B., Ch.B., M.R.C.P.E., to Dr. Andrew Smart's wards; Malcolm Campbell, M.B., Ch.B., F.R.C.S.E., to Dr. D. Berry Hart (Gynæcological Department); Henry Wade, F.R.C.S.Edin., to Mr. Caird's Ward. The following appointments were made for the six months from October 1st:—Resident Physicians—J. W. Keay, M.B., Ch.B., to Professor Sir T. R. Fraser; R. Balfour Barnetson, M.B., Ch.B., to Professor Simpson; W. Sibbald Robertson, M.B., Ch.B., to Dr. Smart; George M'Call Smith, M.B., Ch.B., to Dr. James. Resident Surgeons—Robert Veitch, M.B., Ch.B., to Professor Chiene, C.B.; William J. Fraser, M.B., Ch.B., to Mr. Caird. Clinical Assistants—Robert A. Chambers, M.B., Ch.B., to Professor Sir T. R. Fraser; Lindsay S. Milne, M.B., Ch.B., to Professor Greenfield; L. H. I. Bell, M.B., Ch.B., to Professor Chiene, C.B.; Robert J. Mackessack, M.A., M.B., Ch.B., to Dr. James; Hugh A. Stewart, M.B., Ch.B., to Dr. George A. Gibson; John A. H. Duncan, M.D., F.R.C.S.Edin., (senior), to Dr. M'Kenzie Johnston; John M. Bowie, M.D., etc., to Dr. W. Allan Jamieson; William M'Lachlan, M.B., Ch.B., to Dr. Russell (medical waiting-room); Frederick Porter, M.B., C.M., to Dr. Lovell Gulland (medical waiting-room); Harold Downes, L.R.C.P.&S.Edin., to Dr. Boyd (medical waiting-room). Resident Medical Officer at the Convalescent House, Murrayfield—Frank Inglis Dawson, M.B., Ch.B.

**"The Hospital" Institutional Library.**

**"Hospitals and Asylums of the World." 4 Vols., with a Portfolio of Plans, complete £12 12s.**

**"Burdett's Hospitals and Charities, 1904." 5s. net.**

**"Burdett's Official Nursing Directory." 3s. net.**

**"Cottage Hospitals, General, Fever, and Convalescent." 10s. 6d.**

**"Uniform System of Accounts for Hospitals and Public Institutions." New Edition. 4s. net.**

**"Hospital Expenditure: The Commissariat." 2s. 6d.**

**"A Legal Handbook for the Use of Hospital Authorities." 1s. 6d.**

**"The Cottage Hospital Case Book or Register of Patients." 10s. and 12s. 6d.**

**All these are published by the Scientific Press, Ltd., and may be obtained through any bookseller, or direct from the publishers, 28 and 29 Southampton Street, Strand, London, W.C.**

**Medical Appointments.**

An EXAMINATION OF CANDIDATES for entry into the Medical Department of the Royal Navy will be held on 14th November next and following days at Examination Hall, Thames Embankment.

The forms to be filled up by Candidates will be supplied on application to the Medical Director-General.

Admiralty,

30th September, 1904. 18 Victoria Street, S.W.

**A HANDBOOK FOR NURSES.**

By Dr. J. K. Watson.

5/- net. 5/4 post free.

**PRACTICAL GUIDE TO SURGICAL BANDAGING AND DRESSINGS.**
By W. Johnson Smith, F.R.C.S.
2/- post free.

**THE ART OF MASSAGE.**

By A. Creighton Hale.

*6/- post free.*

**HOME NURSING OF SICK CHILDREN.**

By M. L. Moberly.

*1/- post free.*

**ON PREPARATION FOR OPERATION IN *PRIVATE HOUSES.***

By Stanmore Bishop, F.R.C.S.

7d. post free.

**THE EXAMINATION OF THE URINE.**
*By Dr. J. K. Watson.*
1/- net. 1/1 post free.

**A CASE BOOK FOR PRIVATE NURSING FOR DAY AND NIGHT NURSE.**
48 pages. Size of Exercise Book.
3d. net. 4½d. post free.

**MODERN NURSING IN PRIVATE PRACTICE.**
By Sir William Bennett, F.R.C.S.
6d. net. 7d. post free.

**PRACTICAL HINTS ON DISTRICT NURSING.**
By Miss Amy Hughes.
1/- post free.

**POCKET-BOOK OF TEMPERATURE CHARTS.**
17 twelve-hour and 8 four-hour charts, perforated for removal.
6d. net. 7d. post free.

**NURSING NOTES ON MIDWIFERY AND GYNÆCOLOGY.**
By Sister Ross.
2/- net. 2/1 post free.

**THE NURSES' PRONOUNCING DICTIONARY OF MEDICAL TERMS AND NURSING TREATMENT.**
2/- post free.

**THE "SIMPLEX" DISTRICT NURSES' CASE BOOK.**
For Fifty Cases.
6d. net. 7d. post free.

**HOW TO BECOME A CERTIFIED MIDWIFE.**
By E. Appel, M.B.
1/- net. 1/1 post free.

**THE ART OF FEEDING THE INVALID.**
1/6 post free.

**VISIT BOOKS, TIME BOOKS, LOAN BOOKS FOR DISTRICT NURSES.**

**The Scientific Press, Ltd.,**
28 & 29 Southampton St., Strand, London, W.C.

*Publishers and Booksellers of every kind of Nursing Text-Books, Case-Books and Charts.*
**SEND FOR NEW CATALOGUE.**

